# Preventable long-term complications of suprapubic cystostomy after spinal cord injury: Root cause analysis in a representative case report

**DOI:** 10.1186/1754-9493-5-27

**Published:** 2011-10-27

**Authors:** Subramanian Vaidyanathan, Bakul Soni, Peter Hughes, Gurpreet Singh, Tun Oo

**Affiliations:** 1 Regional Spinal Injuries Centre, Southport and Formby District General Hospital, Town Lane, Southport, PR8 6PN, UK; 2 Department of Radiology, Southport and Formby District General Hospital, Town Lane, Southport, PR8 6PN, UK; 3 Department of Urology, Southport and Formby District General Hospital, Town Lane, Southport, PR8 6PN, UK

## Abstract

**Background:**

Although complications related to suprapubic cystostomies are well documented, there is scarcity of literature on safety issues involved in long-term care of suprapubic cystostomy in spinal cord injury patients.

**Case Presentation:**

A 23-year-old female patient with tetraplegia underwent suprapubic cystostomy. During the next decade, this patient developed several catheter-related complications, as listed below: (1) Suprapubic catheter came out requiring reoperation. (2) The suprapubic catheter migrated to urethra through a patulous bladder neck, which led to leakage of urine per urethra. (3) Following change of catheter, the balloon of suprapubic catheter was found to be lying under the skin on two separate occasions. (4) Subsequently, this patient developed persistent, seropurulent discharge from suprapubic cystostomy site as well as from under-surface of pubis. (5) Repeated misplacement of catheter outside the bladder led to chronic leakage of urine along suprapubic tract, which in turn predisposed to inflammation and infection of suprapubic tract, abdominal wall fat, osteomyelitis of pubis, and abscess at the insertion of adductor longus muscle

**Conclusion:**

Suprapubic catheter should be anchored securely to prevent migration of the tip of catheter into urethra and accidental dislodgment of catheter. While changing the suprapubic catheter, correct placement of Foley catheter inside the urinary bladder must be ensured. In case of difficulty, it is advisable to perform exchange of catheter over a guide wire. Ultrasound examination of urinary bladder is useful to check the position of the balloon of Foley catheter.

## Introduction

Early studies on suprapubic cystostomy in patients with neuropathic bladder reported accelerated renal deterioration and lower urinary tract complications, including stones, recurrent infections and blocked catheters. Hackler [1] showed that suprapubic cystostomy maintained for more than five years caused as much renal damage as the intraurethral catheter retained for more than twenty years. Therefore, Hackler recommended that in spinal cord injury patients, suprapubic cystostomy should be used only temporarily after urethral, ureteric or bladder surgery. In contrast, recent investigations in which patients were managed with anti-cholinergics, frequent catheter changes and bladder washing, demonstrated similar morbidity profiles to patients practicing clean intermittent catheterisation [2].

Complications related to the surgical procedure of suprapubic cystostomy are well documented [3,4]. But there is scarcity of literature on safety issues involved in long-term care of suprapubic cystostomy in spinal cord injury patients. Review of 118 patients with neurogenic bladder managed with suprapubic cystostomy revealed that some complications occurred frequently [5]. Thirty of 118 patients (25%) developed bladder calculi and urinary leakage through the urethra was present in eleven patients (10%). A few rare and unusual complications of suprapubic cystostomy have been documented. Heterotopic bone formation was observed in the pubic region after suprapubic cystostomy and chronic urine leak [6]. Hourglass deformity of urinary bladder is another very unusual late complication of suprapubic cystostomy in persons with neuropathic bladder. Possible reasons for development of hourglass bladder in spinal cord injury patients are: traction applied to dome of urinary bladder by Foley balloon when suprapubic catheter is taped tightly to anterior abdominal wall for several months; uncoordinated contractions of detrusor muscle; chronic cystitis leading to hypertrophy of bladder wall [7]. Dangle and associates [8] reported a patient, who had a neurogenic bladder secondary to multiple sclerosis, and who was managed with a suprapubic catheter. This patient presented with migration of suprapubic catheter into left ureteral orifice, which resulted in left hydronephrosis and obstructive uropathy. We describe a female patient with tetraplegia in whom series of complications occurred during a decade after undergoing suprapubic cystostomy. Documentation of such cases in medical literature will raise awareness amongst health care providers regarding these adverse events, which may be preventable.

### Case Presentation

A 20-year-old woman sustained fracture dislocation of C-4 and C-5, fracture ribs, right pneumothorax, fracture of right humerus, and C-4 complete tetraplegia in a road traffic accident. She was intubated, ventilated and chest drain was inserted. Tracheostomy was performed and this patient was weaned off the ventilator. Initially, this patient was managed by indwelling urethral catheter. Three years later, suprapubic cystostomy was performed because the indwelling catheter had eroded the roof of urethra. Cystoscopy showed erosion of urethra, which was very patulous. Haygrove sound 8/11 was inserted per urethra. A small midline incision was made in suprapubic region through which Haygrove sound was brought out. A 16 French Foley catheter was then inserted into the urinary bladder. Suprapubic catheter was changed once a month.

About a year after undergoing suprapubic cystostomy, the suprapubic catheter came out while a health professional tried to give bladder washout. A sterile catheter was not available and then suprapubic tract had closed. Therefore, a catheter was inserted per urethra. Subsequently, suprapubic cystostomy was performed after introducing a semicircular bougie per urethra and through the dome of urinary bladder and then to the suprapubic region. An 18 French silicone Foley catheter was tied to the tip of the bougie with nylon thread. The 18 French, Foley catheter was gently guided in to the urinary bladder while the bougie was slowly withdrawn.

After twelve months, this patient experienced frequent blockages of catheter. Therefore, flexible cystoscopy was performed. Suprapubic catheter was found to be in proper place. There was no stone; no tumour. Mucosa of urinary bladder was congested. Suprapubic catheter was changed every month in spinal injuries centre for next two years. Then onwards, the catheter was changed in the patient's home by a District Nurse every four weeks.

Fourteen months later, this patient started bypassing every night. Flexible cystoscopy was performed. Urethra was patulous. There was erosion of urethra. The urinary bladder was of small capacity. The tip of suprapubic catheter was located in the urethra. The catheter had gone through patulous bladder neck and had entered the urethra. This patient's carers were taught how to anchor the catheter in the suprapubic region using BioDerm catheter holder. This patient was prescribed oxybutynin modified-release 10 mg once a day. Community health professionals changed suprapubic catheter in the patient's home.

**Figure 1:**
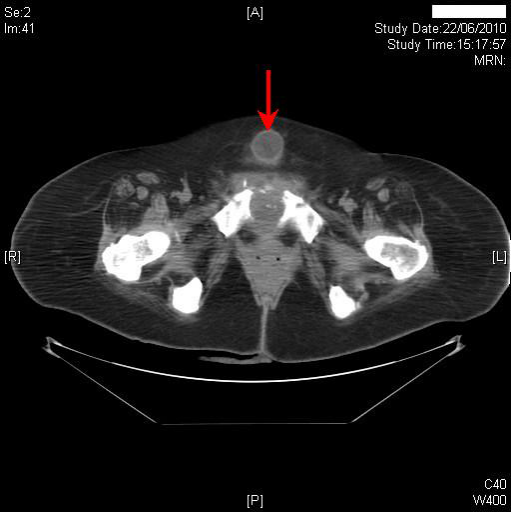
**Computed tomography of abdomen revealed the suprapubic catheter lying outside the bladder**. The balloon (red arrow) and tip of the catheter were located in the subcutaneous adipose layer superficial to the lower part of the rectus sheath.

Two years later, this patient came to spinal unit with history of swelling in the suprapubic region. Clinical examination revealed that the balloon of suprapubic catheter was lying under the skin. A Foley catheter was inserted into the urinary bladder through suprapubic tract. Four months later, the suprapubic catheter inserted by a community health professional, was found to be lying subcutaneously outside the urinary bladder. There was no dressing to anchor the catheter. The catheter was not fixed to skin with any device such as Cath Grip (BioDerm Inc. Largo, Florida 33773, USA). Computed tomography revealed the suprapubic catheter was lying outside the bladder. (Figure 1) The balloon and tip of the catheter were located in the subcutaneous adipose layer superficial to the lower part of the rectus sheath. A month later, this patient started getting small amount of bloody discharge around suprapubic catheter. Ultrasound examination of kidneys revealed normal echotexture to the right kidney, which measured 11.4 cm with good cortical medullary differentiation and cortical depth. An 8 mm calculus was noted in the lower pole calyx of the right kidney. There was no evidence of hydronephrosis. The echotexture of left kidney was normal; left kidney measured 11.3 cm with good cortical medullary differentiation and cortical depth. There was no evidence of hydronephrosis.

**Figure 2:**
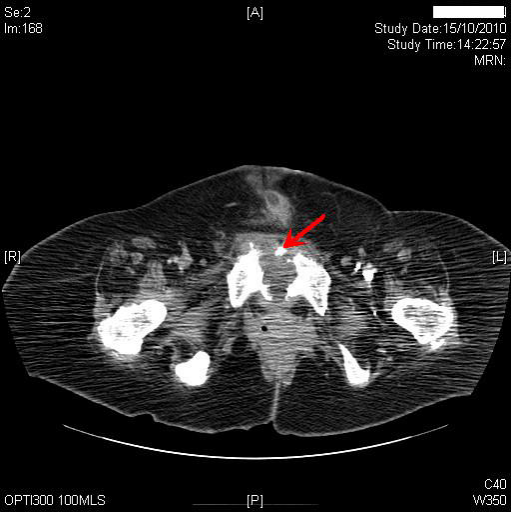
**Computed tomography of abdomen, which was performed four months later, revealed erosion of symphysis pubis with a few bony fragments in the joint space (red arrow)**.

**Figure 3:**
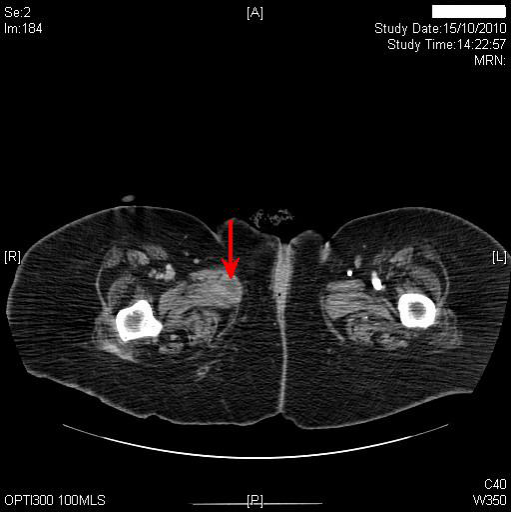
**Computed tomography of abdomen, performed on the same day as Figure 2, revealed a collection measuring 2.6 cm × 2.2 cm at the site of insertion of the right adductor muscle (red arrow)**.

About three months later, patient's carers noticed seropurulent discharge from under-surface of pubis in addition to similar discharge from suprapubic cystostomy site. Computed tomography, revealed the suprapubic catheter balloon to be lying within the empty urinary bladder. There was some midline soft tissue stranding adjacent to the lower abdominal wall muscles at the site of insertion of the suprapubic catheter. There was no evidence of large collection around the suprapubic catheter. More significantly, there were a couple of new small locules of gas in the previously seen small fluid collection (3.4 × 1.6 cm), which was related to the symphysis pubis anteriorly and separate from the suprapubic catheter site. There also appeared to be some erosion of the symphysis pubis with a few bony fragments in the joint space. (Figure 2) Small fluid collection was seen extending down along the proximal right adductor muscle. At the site of insertion of the adductor muscle the collection was 2.6 × 2.2 cm (Figure 3).

Wound swab showed heavy growth of *Streptococcus milleri*, light growth of Coliform species and scanty growth of Proteus species. This patient was prescribed several courses of antibacterial agents based on microbiology reports-Amoxicillin, then Metronidazole, Amoxicillin with Clavulanic acid, then Amoxicillin, followed by Trimethoprim.

**Figure 4:**
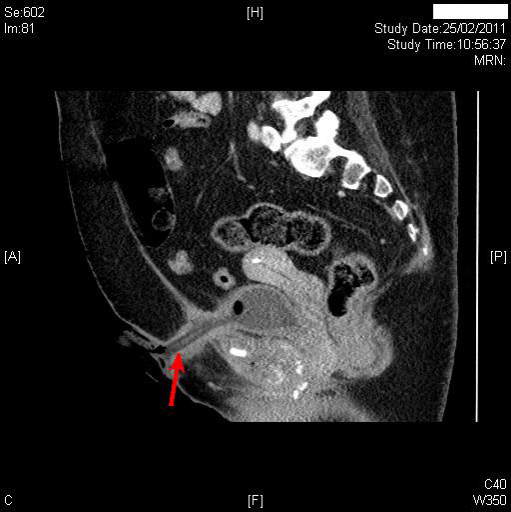
**Sagittal reconstruction of Computed tomography, performed eight months after first CT, revealed persistent soft tissue thickening along the tract of the suprapubic catheter with mild localised inflammation in the abdominal wall fat (red arrow)**. This soft tissue thickening showed some enhancement suggesting chronic inflammation.

Follow-up Computed Tomography performed eight months after the first CT, revealed persistent soft tissue thickening along the tract of the suprapubic catheter with mild localised inflammation in the abdominal wall fat. (Figure 4) This soft tissue thickening showed some enhancement suggesting chronic inflammation. The suprapubic catheter tract was abutting the area of inflammation extending from the site of osteomyelitis at the symphysis pubis; there was a small fluid collection 1.7 cm in diameter containing a small pocket of gas immediately adjacent to the right side of the catheter tract posteriorly. There was persistent inflammatory soft tissue thickening at the symphysis pubis, although bony destruction did not appear to have significantly extended since the previous examination. There were pockets of gas within this and there was an abscess at the right adductor insertion extending for a small distance along the fibres of the right adductor muscle. This collection was 3.8 cm in diameter and had increased since the previous examination.

**Figure 5:**
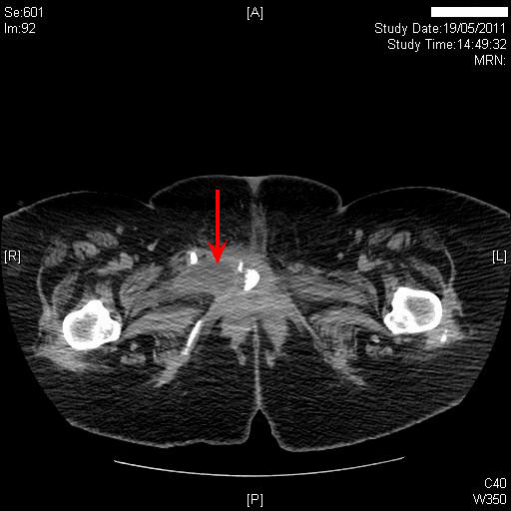
**Computed tomography, performed eleven months after first CT, revealed collection of fluid surrounding the right adductor muscle measuring about 2.7 × 4.8 cm in maximum transverse dimensions (red arrow)**.

**Figure 6:**
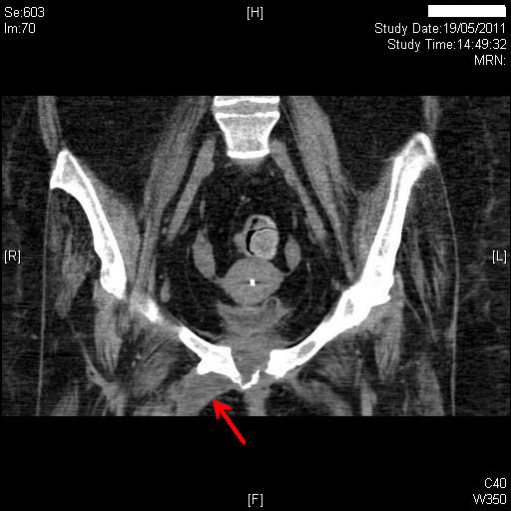
**Coronal reformatted image of Computed tomography, performed eleven months after first CT, revealed collection of fluid surrounding right adductor muscle (red arrow)**.

Computed tomography, performed eleven months after first CT revealed a slight increase in the amount of the previously depicted collection surrounding the adductor muscle measuring about 2.7 × 4.8 cm in maximum transverse dimensions as compared to 2.7 × 3.8 cm previously. (Figures 5 and 6) At the symphysis pubis, there was also increase in the soft tissue component of the symphysis pubis osteomyelitis. The inflammatory soft tissue lesion had slightly increased in size; however, there was almost complete resorption of the previously depicted air loculi. Inflammatory processes still were seen continuous with the thickened suprapubic cystostomy tract. No evidence of newly developed collections or soft tissue abnormalities. A few bilateral subcentimetric inguinal lymph nodes were still noted. There was no evidence of pelvic collections.

Currently, this patient continues to manage her bladder by suprapubic cystostomy. A size 24 French, 30 ml balloon Bard Biocath Hydrogel coated Foley catheter is being used for suprapubic cystostomy. There is no discharge from under-surface of pubis. The suprapubic cystostomy site looks healthy with no evidence of inflammation.

## Discussion

Reported complications during long-term care of suprapubic cystostomy include urine infection, stones in urinary bladder, renal calculi, haematuria, neoplastic changes occurring in urinary bladder, at the site of cystostomy or in the suprapubic tract [9,10]. Dislodgement of catheter requiring re-operation, or migration of tip of catheter through patulous bladder neck into urethra probably do occur but have not been publicised in medical literature. Infection compounded by chronic inflammation involving suprapubic tract, abdominal wall fat, and pubic bone with abscess formation at the site of insertion of adductor longus have not been described at all. We report these complications so that awareness is created amongst medical administrators and health professionals, who look after spinal cord injury patients with suprapubic cystostomy. Safety issues related to long-term care of suprapubic cystostomy merit the attention of health care providers in the community so that the quality of life of persons with profound disability such as our patient is not compromised by adverse events, which may be preventable. From the point of view of hands on clinical practice, this case illustrates the need for meticulous attention while changing suprapubic catheter so that the balloon of Foley catheter is not inflated outside urinary bladder. Further, suprapubic catheter needs to be anchored securely in order to prevent accidental dislodgement from urinary bladder or, downward migration of the tip of catheter through patulous bladder neck.

We have been anchoring the catheter in suprapubic region with CathGrip (made by BioDerm Inc. Largo, Florida 33773, USA), as CathGrip ensures satisfactory anchorage of all sizes of suprapubic catheter. Further, there is no sharp edge anywhere and CathGrip is skin-friendly. Anchoring the suprapubic catheter prevents migration of catheter into urethra as well as dislodgement due to inadvertent pull on the catheter. If the suprapubic catheter had been anchored securely in this patient, the catheter would not have come out and the patient would not have required re-operation. Other products such as GRIP-LOK™ Universal Foley Catheter Securement, and Statlock Catheter Stabilization device, which is manufactured by Bard Medical, are available for securing and stabilising Foley catheters. Grip-Lok allows a degree of rotational, and limited horizontal, movement, although such movement can be adjusted by applying the Velcro in a firmer fashion. Statlock helps eliminate catheter kink and enhances patient comfort.

While changing suprapubic catheter, health professionals should ensure that the balloon of Foley catheter is located within the urinary bladder. When a suprapubic catheter is taken out, we measure the length of Foley catheter which has been inside starting from the level of skin. The same length of a new Foley catheter is then inserted. If there is any doubt, we perform ultrasound scan of urinary bladder to check the position of the balloon of Foley catheter. There can be difficulty in changing a suprapubic catheter in obese individuals who have a large, pendulous belly as well as a small capacity urinary bladder. In obese individuals with a small bladder, the distance between the skin of abdominal wall and urinary bladder may be considerable. When suprapubic track is long and tortuous for safe change of catheter in a routine manner, we perform exchange of catheters over a 0.032 guide wire [11]. We use a Folysil catheter (manufactured by Coloplast Ltd, Peterborough PE2 6BR, United Kingdom), which has a terminal hole. The terminal hole in Folysil catheter facilitates insertion of a new catheter over a guide wire. This technique has proved to be a reliable method for changing suprapubic catheters in obese patients.

In this patient, the catheter was positioned outside the urinary bladder at least on two occasions. Incorrect placement of suprapubic catheter resulted in chronic leakage of urine along suprapubic tract and infection spreading to suprapubic tract, abdominal wall fat, pubic symphysis and adductor longus muscle. Microbiology of discharge showed growth of *Streptococcus milleri*. *Streptococcus milleri *group organisms are commensals of the oral cavity and of the gastrointestinal tract. *Streptococcus milleri *group organisms are notorious causes of pyogenic, invasive infections, and have been found in head and neck abscesses, bacteraemia with endocarditis, liver abscess, thoracic empyema, brain abscess, and spinal epidural abscess [12,13]. Patients with underlying medical conditions, such as cirrhosis, diabetes mellitus, and malignancies, are predisposed to invasive infections with *Streptococcus milleri *[14]. Our patient did not have cirrhosis, diabetes mellitus or malignancy, but was suffering from tetraplegia due to spinal cord injury. Spinal cord injury has been shown to affect adversely the immunological system. Riegger and associates [15] showed that spinal cord injury was associated with an early onset of immune suppression and secondary immune deficiency syndrome. Infection of suprapubic cystostomy site with *Streptococcus milleri *is very unusual. Search in PubMed for "*Streptococcus milleri*" and either "suprapubic cystostomy" or "spinal cord injury" showed no publication. In our clinical practice of spinal cord medicine during the past three decades, this is the first case of infection of suprapubic cystostomy site with *Streptococcus milleri*.

In our patient, the seropurulent discharge drained freely along the suprapubic tract into the dressing applied around suprapubic catheter. This patient's carers applied new dressing every day. This patient received several courses of antibiotics and suprapubic catheter was changed at fortnightly interval. Therefore, this patient did not require any surgical intervention to facilitate drainage of discharge arising from infected pus.

Search in PubMed for "suprapubic cystostomy", and "osteomyelitis" yielded no publication. But osteomyelitis of pubis has been reported following lower urinary tract surgery [16]. Andonian and associates [17] described a 66-year-old diabetic man, who presented with acute incapacitating pelvic pain six weeks after radical prostatectomy. Symphysis pubis biopsy showed chronic osteomyelitis, and culture grew *Pseudomonas aeruginosa*. To the best of our knowledge, this is the first case report of osteomyelitis of pubis in a spinal cord injury patient, which occurred due to misplacement of suprapubic catheter and chronic leakage of urine along suprapubic track. An interesting feature of this case is isolation of *Streptococcus milleri *from suprapubic discharge at least on two occasions. Previous reports of osteomyelitis of pubis following radical prostatectomy showed infection due to coliform organisms. We have not seen a spinal cord injury patient, who had uneventful suprapubic cystostomy, developing osteomyelitis of pubis in our clinical practice spanning more than three decades. Therefore, we believe that our patient developed osteomyelitis as a consequence of chronic leakage of urine because of misplaced catheter.

## Conclusion

Learning points from this case: Suprapubic catheter should be anchored securely to prevent migration of the tip of catheter into urethra and accidental dislodgment of catheter. While changing the suprapubic catheter, health professional should ensure correct placement of Foley catheter inside the urinary bladder and avoid inflating the balloon outside the bladder. In case of difficulty, it is advisable to perform exchange of catheter over a guide wire. Ultrasound examination of urinary bladder is really very useful to check the position of the balloon of Foley catheter.

## Consent

Written informed consent was obtained from the patient for publication of this Case report and accompanying images. A copy of the written consent is available for review by the Editor-in-Chief of this journal.

## Competing interests

The authors declare that they have no competing interests.

## Authors' contributions

SV conceived the idea and wrote the manuscript. PH reported medical images. BMS was the consultant in charge of the patient. GS performed suprapubic cystostomy. TO participated in care of this patient. All authors read and approved the final manuscript.
